# Core decompression and bone marrow aspirate injection in avascular necrosis of the femoral head

**DOI:** 10.1097/MD.0000000000042012

**Published:** 2025-05-02

**Authors:** Oğuzhan Şamil Erciyes, Emre Gültaç, Fatih İlker Can, Umut Canbek, Cem Yalin Kilinç, Nevres Hürriyet Aydoğan

**Affiliations:** a Department of Orthopedics and Traumatology, Muğla Sitki Koçman University Training and Research Hospital, Muğla, Turkey.

**Keywords:** avascular necrosis, biological therapy, bone marrow aspirate injection, femoral head

## Abstract

To evaluate the clinical and radiological outcomes of core decompression combined with bone marrow aspirate injection (BMAI) in patients with femoral head avascular necrosis (AVN) before articular surface collapse. Twenty-two patients with AVN underwent core decompression and BMAI. The patients were followed for at least 12 months. Radiological assessments were performed using plain radiography and magnetic resonance imaging, and staging was conducted based on the Association Research Circulation Osseous system. Clinical outcomes were evaluated using the Harris Hip Score and Merle d’Aubigne-Postel scoring systems. At the 12-month follow-up, hip function scores showed significant improvement. The mean Harris Hip Score increased by 23 points (*P* < .001), and the mean Merle d’Aubigne-Postel score increased by 1 point (*P* < .001). Radiologically, all stage I lesions regressed to stage 0 (*P* < .001). Among stage II lesions, 83% remained stable, while progression was observed in 17%. 75% of the stage IIIA lesions showed radiological progression. Core decompression combined with BMAI effectively halts the progression of early-stage AVN, preserving joint integrity and delaying the need for total hip arthroplasty. Further studies are warranted to explore the efficacy of biological therapy in treating femoral head AVN.

## 1. Introduction

Avascular necrosis (AVN) of the femoral head is a debilitating condition characterized by ischemic bone necrosis due to compromised blood flow. It predominantly affects individuals between 30 and 50 years of age, leading to joint collapse and osteoarthritis if left untreated. Early diagnosis and intervention are critical, as AVN’s natural progression often results in severe pain, loss of function, and the need for total hip arthroplasty.^[1]^

The etiology of AVN includes trauma, prolonged corticosteroid use, alcohol consumption, and idiopathic causes. Despite these known factors, the precise mechanisms remain unclear, making treatment challenging.^[2]^ Conventional treatments, including pharmacological agents and physical therapy, have shown limited success in halting disease progression. Consequently, surgical interventions have become the primary focus for managing AVN, particularly in its early stages.

Core decompression is a minimally invasive procedure aimed at reducing intraosseous pressure and promoting revascularization in necrotic areas. Recent advancements have integrated biologic therapies, such as bone marrow aspirate injection (BMAI), which delivers mesenchymal stem cells (MSCs) and growth factors directly to the lesion site. This combination is hypothesized to enhance bone repair and regeneration by improving vascularity and osteogenesis.^[3]^

Several studies have supported the efficacy of core decompression with BMAI in delaying or preventing the progression of AVN. For instance, Wang et al demonstrated that patients treated with core decompression and BMAI had better clinical and radiological outcomes compared to those undergoing core decompression alone.^[4]^ Moreover, Hernigou et al (2018) reported long-term benefits of MSC-enriched therapies in reducing the need for arthroplasty in early-stage AVN.^[5]^

The purpose of this study is to evaluate the clinical and radiological outcomes of core decompression combined with BMAI, with an emphasis on its efficacy in different stages of AVN. By analyzing these outcomes, this study aims to contribute to the growing evidence supporting biologically augmented surgical interventions as a standard approach for managing AVN.

## 2. Methods

### 2.1. Study design and participants

This study was administered in compliance with the ethical doctrines presented in the Declaration of Helsinki and received approval from the institutional ethical board (07.11.2024/1835). The preoperative and postoperative 12-month clinical and radiological data of patients diagnosed and treated for avascular necrosis (AVN) of the femoral head at our clinic was analyzed. Preoperative and postoperative anteroposterior pelvis radiographs and hip magnetic resonance imaging (MRI) scans were assessed, with staging classified according to the Association Research Circulation Osseous (ARCO) system. Demographic and etiological group distributions were recorded, and changes in staging between the preoperative and postoperative periods were evaluated concerning the preoperative stage, etiology, and patient age.

### 2.2. Clinical evaluation

Preoperative and postoperative functional outcomes were assessed using the Harris Hip Score (HHS) and Merle d’Aubigné-Postel scores (MDPS), extracted from outpatient clinic records. Preoperative scores were analyzed for their distribution across age, gender, and etiological groups. Changes in scores from preoperative to postoperative assessments were analyzed concerning ARCO staging, stage progression, etiology, and age.

### 2.3. Diagnostic protocol

Patients presenting with hip pain underwent initial evaluation with anteroposterior pelvis radiography following a detailed medical history and physical examination. Radiographic findings such as focal osteopenia, sclerosis, osteolysis, subchondral diffuse osteopenia, and crescent signs were used to identify potential AVN. MRI was used for staging in cases with radiographic evidence of AVN or when the history revealed risk factors such as steroid use, alcohol consumption, hypercoagulability, hemoglobinopathies, or rheumatologic conditions, even in cases of normal radiographs. Patients with inconclusive findings underwent a 2-week conservative follow-up with NSAIDs and activity restriction, followed by MRI if symptoms persisted. For cases with MRI findings suggestive of bone marrow edema, a 45-day period of rest and non-weight-bearing was instituted, with reevaluation using MRI to confirm resolution and exclude AVN.

### 2.4. Surgical and laboratory management

Patients diagnosed with ARCO stages I–II or IIIA AVN, aged < 65 years, without joint surface collapse, and not on ongoing steroid therapy were offered core decompression and bone marrow injection. Preoperative assessments included routine HHS and MDPS. Laboratory evaluations included serum vitamin D, calcium, and phosphorus levels. Replacement therapy was initiated for patients with deficiencies to optimize postoperative bone metabolism.

### 2.5. Surgical procedure

**Figure 1.**
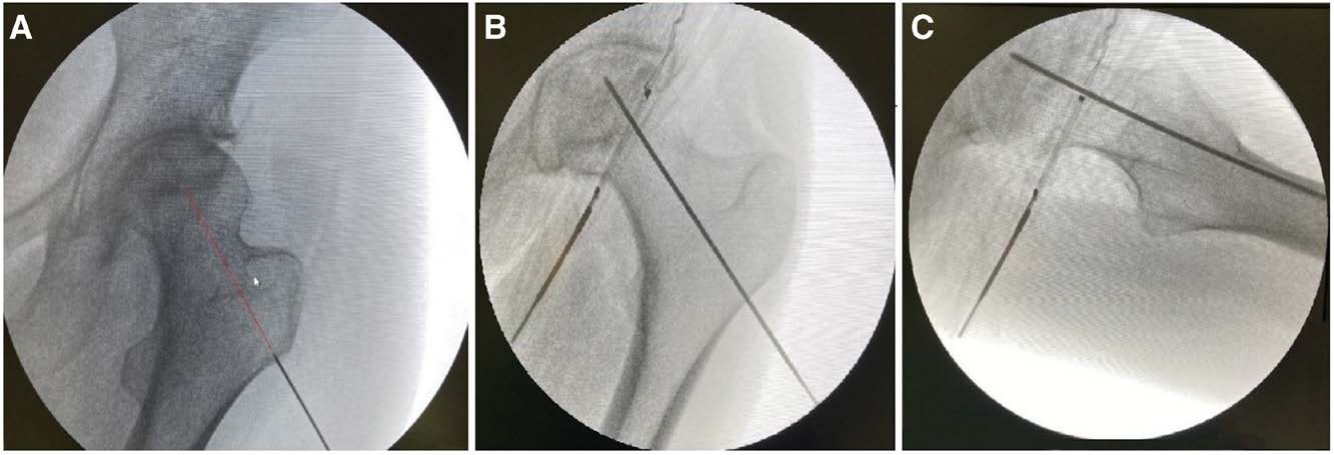
(A) Anteroposterior X-ray view of the avascular femoral lesion. (B) Anteroposterior view of guide K-wire application. (C) Lateral X-ray view of the K-wire application.

All patients were placed in the lateral decubitus position on the radiolucent operation table after spinal anesthesia. The location of the lesion determined in the preoperative planning was targeted and a 2 mm guide wire was advanced percutaneously from the intertrochanteric region under fluoroscopy guidance. While advancing the wire, after reaching the necrotic area after the hard transition zone due to the sclerotic band, the soft area was sensed and the lesion was confirmed with anteroposterior and lateral fluoroscopy imaging (Fig. 1).

**Figure 2.**
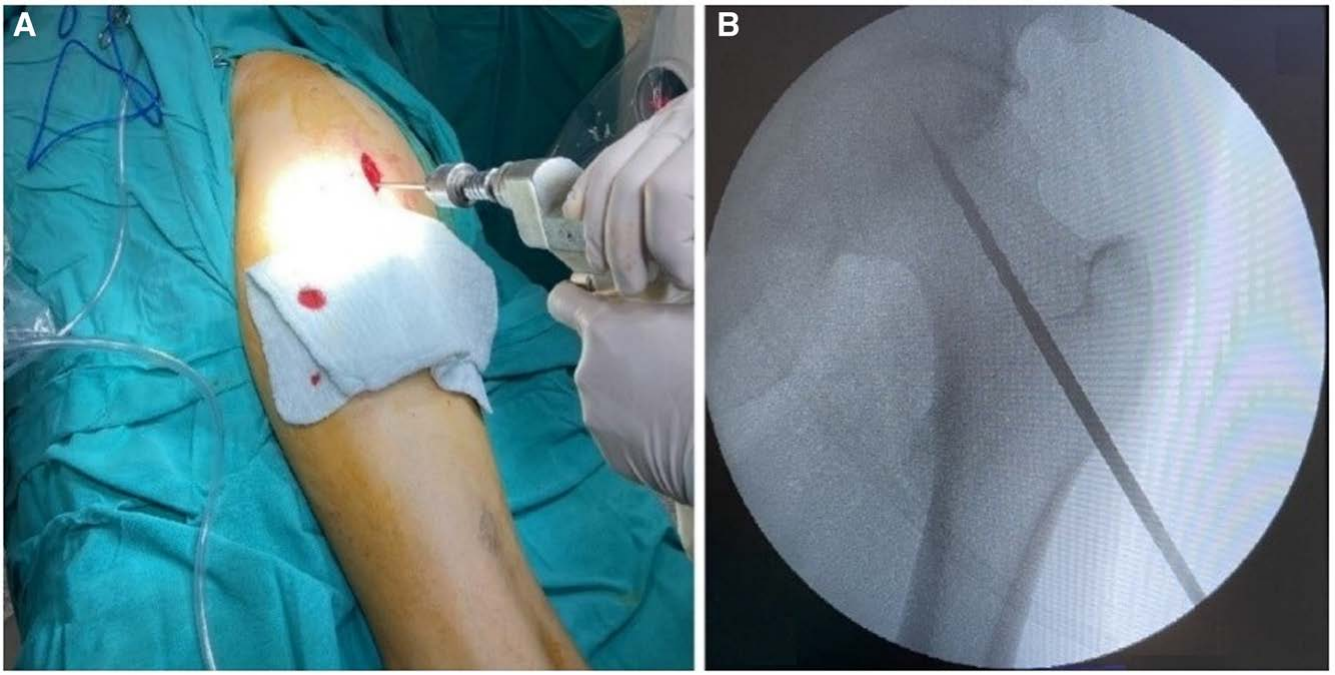
(A) Intraoperative surgical view of the incision site. (B) Anteroposterior X-ray view of the drilling via the guide K-wire.

A 3 to 4 cm longitudinal incision was made from the entry point of the guide wire, the skin and subcutaneous tissues were cut, the tensor fascia lata was separated in a split manner and the bone was reached. The lesion was reached over the guide wire using a 4.5 mm cannulated drill, the tunnel was opened and the core decompression phase was completed (Fig. 2). Bone marrow aspirate injection into the femoral head is performed with the Jamshidi needle that we use for bone marrow aspiration from the iliac crest. Since the thickness of this needle is compatible with the 4.5 mm drill tip, the drill size was also preferred as 4.5 mm.

**Figure 3.**
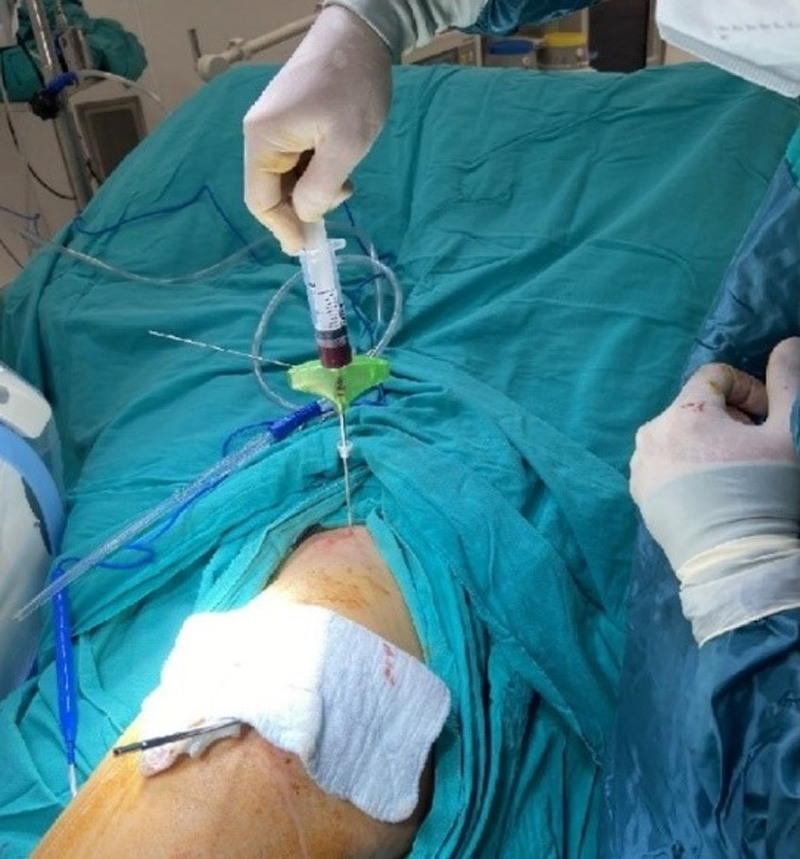
Harvesting the bone marrow aspirate from the iliac crest.

**Figure 4.**
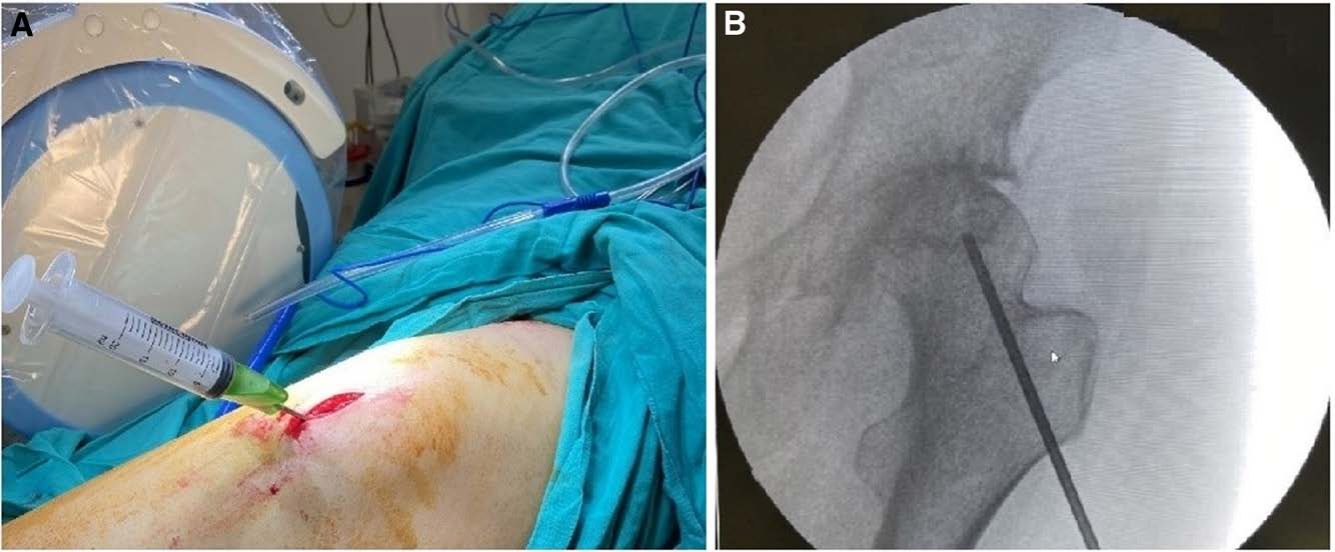
(A) Intraoperative surgical view of the bone marrow aspirate injection. (B) Anteroposterior X-ray view of the bone marrow aspirate injection.

After the core decompression was completed, approximately 10cc of bone marrow aspirate was taken from the posterior spina iliaca superior region of the patients with a bone marrow biopsy needle (Fig. 3). Then, the drill was removed from the opened tunnel, the bone marrow biopsy cannula was placed and the bone marrow aspirate was injected into the lesion (Fig. 4).

After the injection was administered, the tunnel entry point was closed with bone wax, the skin and subcutaneous tissues were sutured and the operation was concluded. Postoperative hip joint range of motion exercises were started for the patients, and 8 weeks of non-weight-bearing mobilization with crutches were explained. Low molecular weight heparin (enoxaparin) was prescribed for 30 days for deep vein thrombosis prophylaxis. After the necessary checks were made in the 8th week postoperative follow-ups, anterior–posterior pelvic radiographs were taken, and weight-bearing on the extremity was allowed.

The patients were followed up at 6 and 12 months postoperatively. At the 12th month follow-up, physical examinations, HSS and MDS scores of the patients were recorded. Radiological progression was examined with MRI, and stage changes, if any, were recorded.

### 2.6. Clinical and radiological assessment

Pre- and postoperative evaluations included HHS and MDPS. Radiological staging was performed using ARCO criteria, based on pelvic X-rays and MRI.

### 2.7. Statistical analysis

Statistical analysis was performed using the SPSS version 18.0 software (SPSS Inc., Chicago, IL). Shapiro–Wilk test was used in normal distribution analysis, and nonparametric tests were used to observe that the data were not normally distributed except for the age group. Descriptive tests were used in the analysis of demographic data, and the Mann–Whitney *U* test was used to compare clinical scores. Kruskal–Wallis test was used to investigate the difference between HHS and MDS between different stages before and after surgery, and Pairwise comparison test was used for detailed analysis, and Wilcoxon signed-rank test was used to examine the effect of applied treatments on these scores. *P* < .05 was considered significant in data analysis.

## 3. Results

A total of 22 patients underwent surgery, comprising 14 males (63%) and 8 females (37%) with a median age of 44 years (range: 25–60 years). No statistically significant relationship was observed between age and preoperative disease stage (*P* > .05). Among the etiological factors, 7 patients reported a history of steroid use, and 1 patient had experienced a traumatic hip dislocation a year prior. The remaining 14 patients were categorized as idiopathic, with no identifiable risk factors such as trauma, hemoglobinopathy, thrombophilia, or chronic alcoholism.

All patients presented with hip pain, which was frequently described as radiating from the hip to the thigh, accompanied by gait abnormalities and restricted motion. Functional assessment showed significant limitations in hip flexion and abduction due to pain. Postoperative follow-up at 12 months revealed that 36% of patients reported complete pain resolution, 31% experienced significant pain reduction, 9% noted no change, and 18% reported worsening pain.

Functional outcomes were evaluated using the HHS and the MDPS system. Preoperative median HHS was 55 (range: 35–65), improving to a median of 78 (range: 40–100) postoperatively (*P* < .001). Similarly, preoperative MDPS scores improved from a median of 3.7 (range: 3–4.3) to 4.7 (range: 2.3–6) postoperatively (*P* = .001). Subgroup analysis revealed that improvements in HHS and MDPS scores were statistically significant in stages I and IIIA patients but not between stages II and IIIA.

The ARCO classification showed that 8 patients initially in stage I regressed to stage 0 postoperatively (*P* < .001). However, among stage II patients, 5 showed no change, and 1 progressed to stage IIIB. In stage IIIA patients, 4 progressed to stage IIIB and 2 to stage IV. Overall, the changes in ARCO stages were significant (*P* = .002), with posttreatment differences observed between stages I and II (*P* = .001) and stages I and III (*P* = .000), but not between stages II and III (*P* = .051).

No surgical complications such as thrombophlebitis, pulmonary embolism, intertrochanteric fractures, or wound infections were noted. Total hip arthroplasty was performed in patients with symptomatic advanced coxarthrosis.

## 4. Discussion

Our study demonstrates the efficacy of core decompression combined with BMAI in managing AVN of the femoral head, particularly in early-stage lesions. Functional improvements, as evidenced by significant increases in HHS and MDPS, align with prior research underscoring the role of biological augmentation in improving outcomes for AVN patients.^[5]^

The combination of the core decompression technique with bone marrow injection containing autologous MSCs has become popular in early-stage patients, with studies reporting successful results published in recent years.^[6–11]^ Hernigou first suggested the injection of autologous mesenchymal cells into the necrotic area in 2002. The authors reported the restorative effect of cells taken from the patient’s iliac crest and then reinjected into the necrotic area using the traditional core decompression technique.^[12]^

Our findings corroborate existing studies highlighting the role of MSCs in promoting bone regeneration. For example, Wang et al demonstrated superior clinical and radiological outcomes with core decompression and BMAI compared to core decompression alone, attributing this to the MSCs’ osteogenic potential.^[4]^ Similarly, Migliorini et al emphasized the importance of combining mechanical decompression with biological therapies to enhance vascularity and osteogenesis.^[13]^

The efficacy of the intervention was notably higher in ARCO stage I lesions, where a regression to stage 0 was observed in all cases. This finding is consistent with theories that early intervention can capitalize on the greater viability of bone and vascular structures in the early stages.^[1]^ In contrast, while 83% of stage II lesions remained stable, progression in 17% and the notable radiological progression in 75% of stage IIIA lesions underscore the challenges posed by advanced AVN. In a 2015 study involving 29 patients, Pietro et al reported successful treatment in 100% of patients with stage I necrosis and 87.5% of those with stage II necrosis. However, treatment failure occurred in 12.5% of stage II patients and in all patients with stages III and IV necrosis.^[14]^ Similarly, in a study by Joon Sun et al, core decompression combined with bone marrow-derived stem cell therapy was applied to 53 hips, including 30 at ARCO stages I–II and 23 at ARCO stage III. After a 4-year follow-up, total hip arthroplasty (THA) was required in 6 (20%) of the stages I–II hips and 10 (40%) of the stage III hips.^[15]^ In a study, Yoshioka et al conducted a 10-year follow-up of 80 patients treated with core decompression. Among these, 12 were at ARCO stage I, 31 at stage II, 32 at stage III, and 5 at stage IV. THA was required in 27 patients (34%), with an 18% incidence in the precollapse (stages I–II) group and 58% in the collapse (stages III–IV) group.^[16]^ In our study, 12-month postoperative MRI findings indicated almost complete recovery in all 8 ARCO stage I patients. Among the 6 ARCO stage II patients, no progression was detected in 5, while 1 patient progressed to stage IIIB. Of the 8 patients with ARCO stage IIIA, 2 progressed to stage IV and required THA, 4 progressed to stage IIIB, and 2 remained unchanged. These data correspond to a 75% progression rate in stage IIIA patients. Consistent with previous findings, our results underscore that lower ARCO stages at the time of treatment with core decompression and bone marrow injection are associated with higher success and survival rates.

The observed improvements are likely attributable to the dual mechanisms of core decompression and BMAI. Core decompression reduces intraosseous pressure, improving local circulation.^[17]^ BMAI contributes to this by delivering MSCs and growth factors, which play a critical role in angiogenesis and osteogenesis.^[5]^

### 4.1. Limitations and future directions

While this study adds to the growing evidence supporting biologically augmented surgical interventions, it is not without limitations. First, the sample size of 22 patients is relatively small, limiting the generalizability of the results. Additionally, the follow-up period of 12 months may not fully capture the long-term outcomes of the intervention. Larger, multicenter trials with extended follow-ups are needed to validate these findings. Moreover, the lack of significant improvement among stage II patients and progression in stage IIIA patients warrants further investigation into the underlying factors. Stratification based on etiology, such as corticosteroid use or alcohol consumption, could provide valuable insights into differential responses to treatment.^[17]^

## 5. Conclusion

In conclusion, core decompression combined with BMAI is a promising intervention for early-stage AVN, offering significant functional and radiological improvements. However, its efficacy diminishes with disease progression, underscoring the importance of early diagnosis and treatment. Future research should focus on refining patient selection criteria and exploring adjunctive therapies to enhance outcomes in advanced stages of AVN.

## Author contributions

**Conceptualization:** Oğuzhan Şamil Erciyes.

**Data curation:** Emre Gültaç, Fatih İlker Can.

**Formal analysis:** Emre Gültaç, Cem Yalin Kilinç.

**Investigation:** Cem Yalin Kilinç.

**Methodology:** Oğuzhan Şamil Erciyes, Fatih İlker Can, Umut Canbek.

**Software:** Fatih İlker Can, Umut Canbek.

**Supervision:** Cem Yalin Kilinç.

**Writing – original draft:** Oğuzhan Şamil Erciyes, Emre Gültaç.

**Writing – review & editing:** Nevres Hürriyet Aydoğan.
